# The N Terminus of Orf Virus-Encoded Protein 002 Inhibits Acetylation of NF-κB p65 by Preventing Ser^276^ Phosphorylation

**DOI:** 10.1371/journal.pone.0058854

**Published:** 2013-03-11

**Authors:** Zhangyong Ning, Zewei Zheng, Wenbo Hao, Chaohui Duan, Wei Li, Yuanyuan Wang, Ming Li, Shuhong Luo

**Affiliations:** 1 College of Veterinary Medicine, South China Agricultural University, Guangzhou, People's Republic of China; 2 Institute of Antibody Engineering, School of Biotechnology, Southern Medical University, Guangzhou, People's Republic of China; 3 Laboratory of Clinical Immunology, The Sun Yat-Sen Memorial Hospital, Sun Yat-Sen University, Guangzhou, People's Republic of China; Institut national de la santé et de la recherche médicale – Institut Cochin, France

## Abstract

Orf virus-encoded protein 002 (ORFV002) inhibits NF-κB signaling pathway by decreasing the acetylation of NF-κB-p65 through interference of NF-κB p65′s association with NF-κB p300. However, the precise mechanism of how ORFV002 interferes with the NF-κB p65/p300 association is still unknown. Due to similarities of the amino acid sequences of ORFV002 and the adenovirus type 12 (Ad12) E1A protein (E1A-12), we hypothesized that the N-terminal 52 amino acids of ORFV002 might play an important role in this inhibition and constructed several in-frame fusions of *ORFV002* to an enhanced green fluorescent protein (EGFP) reporter, including C-terminal and N-terminal deletion mutants of *ORFV002*. When the N-terminus of ORFV002 was absent, the localization of ORFV002 shifted mainly from the nucleus to the cytoplasm, and it's inhibition of NF-κB transactivation was lost. NF-κB p65 Lys^310^ acetylation and Ser^276^ phosphorylation were detected in co-transfection experiments with NF-κB p65 and ORFV002 or its mutants with, or without, the N-terminal region. The results showed that the N-terminus of ORFV002 plays a crucial role in inhibiting both the acetylation and phosphorylation of NF-κB p65. Further investigation indicated that ORFV002 and its C-terminal deletion mutants interfered with NF-κB p65 (Ser^276^) phosphorylation induced by mitogen- and stress-activated protein kinase-1 (MSK1) and the interaction between NF-κB p65 and MSK1. Since phosphorylated NF-κB p65 recruits transcriptional co-activators such as p300 and CBP, we concluded that the N-terminus of ORFV002 inhibits acetylation of NF-κB p65 by blocking phosphorylation of NF-κB p65 at Ser^276^.

## Introduction

The orf virus (ORFV), the type species of the *Parapoxvirus* genus, is an epitheliotrophic virus and causative agent of the orf disease, which mainly affects sheep, goats, wild ruminants and humans with a worldwide distribution [1]-[4]. The ORFV genome is 138 kb with G+C content up to 64 percent and it contains 132 putative genes [5], [6]. This parapoxvirus has evolved various strategies including encoding a protein to inhibit activation of the nuclear factors κB (NF-κB) signaling pathway [4]. Several genes of the orf virus including 121 [7], 024 [8] and 002 [4] modulate host immune function by inhibiting the activation of NF-κB. This is one of the strategies that the orf virus uses to escape the host's immunological response and modify the disease process.

Inducible phosphorylation has been reported to occur at multiple NF-κB p65 sites, including Ser^536^ and Ser^276^, leading to the changes in NF-κB activation [9]. The human adenovirus type 12(Ad12) E1A protein (E1A-12) inhibits phosphorylation of NF-κB p65 at Ser^276^ and causes the loss of DNA binding and transactivation to activate major histocompatibility complex class I expression [10]. Further study found that the last 66 amino acids of the N-terminus of adenovirus type 12 E1A were as effective as the wild-type E1A protein in preventing phosphorylation of NF-κB p65 at Ser^276^ and inhibiting major histocompatibility complex class I expression [11]. Our previous report indicated that the *ORFV002* gene inhibits the NF-κB signaling pathway by decreasing TNF-α. Furthermore, the wild-type virus induced acetylation of NF-κB p65 by interfering with the NF-κB p65/p300 association and not by affecting phosphorylation of NF-κB p65 at Ser^536^ [4]. The precise mechanism of how ORFV002 interferes with the NF-κB p65/p300 association is not clearly understood.

Based on the amino acid sequence analysis we found that ORFV002 shares three putative conserved protein domains with the E1A-12 protein. We hypothesized that the N-terminus of the ORFV002 has a similar function for the phosphorylation of NF-κB p65. Our results confirmed that the N-terminal 52 residues of ORFV002 could inhibit phosphorylation of NF-κB p65 at Ser^276^ blocking the subsequent acetylation of NF-κB p65. This unique property of ORFV002 disables NF-κB transactivation. This is the first report of N-terminus of ORFV002 protein targeting nuclear events by regulating NF-κB transactivation.

## Materials and Methods

### Animal and Cell Preparation

Two 3-year old Han sheep, which were pregnant for 120 days, were obtained from the Center of Laboratorial Animals in South China Agricultural University, China. The ovine experiments were approved by the Institutional Animal Care and Use Committee at South China Agricultural University (Certification Number: CNAS BL0011). The primary ovine fetal turbinate tissues (OFTu) were prepared as previously described with the bovine turbinate cells [12], with some modifications. The sheep were anesthetized, and the fetuses were obtained. The turbinate tissue was cut into small pieces and trypsinized in 0.125% trypsin for 15 minutes at room temperature to settle the cells. Then the trypsin was decanted, and fresh trypsin was added for 30 minutes. The dispersed cells were decanted and saved; the digestive procedure was repeated on the remaining tissues. Dispersed cells collected in these serial digestions were pooled and washed twice in minimal essential medium (MEM), diluted at 5×10^5^ cells/ml of complete medium and seeded at 30 ml per 150 cm^2^ plastic flasks. The complete medium consisted of MEM supplemented with 10% fetal bovine serum (FBS), 100 μg/ml streptomycin, 100 U/ml penicillin, 50 μg/ml gentamicin and 2 mM L-glutamine.

### Plasmid Construction

*ORFV002* coding sequences were synthesized by EZBiolab, Inc. (Westfield, IN, USA), and subcloned into the expression vector pEGFP-N1 (Clontech, Mountain View, CA, USA) to generate the p002EGFP plasmid. DNA sequencing of p002EGFP confirmed the integrity of *ORFV002* coding sequences and in-frame cloning with enhanced green fluorescent protein (EGFP). N-terminal fusions of EGFP to ORFV002 deletion mutants were constructed by PCR in a method similar to that described in [4]; p002EGFP was used as a template to amplify ORFV002-EGFP mutant 1 (Δ1-52, 002GM1 or M1), ORFV002-EGFP mutant 2 (Δ1–94, 002GM2 or M2), ORFV002-EGFP mutant 3 (Δ95–117, 002GM3 or M3), ORFV002-EGFP mutant 4 (Δ53–117, 002GM4 or M4). The primer sequences were synthesized as follows:

#### 002GM1-Fw

5′-ACTAT**AAGCTT**GCCACCATGCTTGCAGCTGGAGCTAGAGC-3′;

#### 002GM1-Rv

5′-ATAACT**GCGGCCGC**TTACTTGTACAGCTCGTCCATGC-3′;

#### 002GM2-Fw

5′- ATCATG**AATTC**GCCACCATGAGAGGAGCAGCTGCATCG-3′;

#### 002GM2-Rv

5′- ATAACT**GCGGCCGC**TTACTTGTACAGCTCGTCCATGC-3′;

#### 002GM3-Fw

5′-GATCAT**GAATTC**GCTAGCCACCATGACTCCTACTTCTCGAGA-3′;

#### 002GM3-Rv

5′- GGT**GGATCC**ATCCTAGCATGAGTAGTGG-3′;

#### 002GM4-Fw

5′-GATCAT**GAATTC**GCTAGCCACCATGACTCCTACTTCTCGAGA-3′;

#### 002GM4-Rv

5′- GGT**GGATCC**ATTAGTGCTCTCACAAGTGC-3′.

The PCR was carried out in a 50 μl reaction volume containing 1 μl of DNA template, 0.4 μM of each primer and 0.5 μl of *Taq* polymerase (Promega Co., Madison, WI, USA) in 10 μl of 5× PCR buffer (10 mM Tris-HCl, and 50 mM KCl and 25 μM MgCl_2_) with 200 μM final concentrations each of dATP, dTTP, dCTP and dGTP, 1 μl of DNA template, 200 μM dATP, dTTP, dCTP, dGTP, 0.4 μM of each primer, 25 μM MgCl2 and 0.5 μl of *Taq* polymerase (Promega Co., Madison, WI, USA). PCR was performed in a thermocycler (GeneAmp PCR 2400, PE Applied Biosystems Corp, Foster, CA, USA) for 30 cycles of denaturation (94°C for 1 minute), annealing (58°C for 30 seconds) and extension (72°C for 1.5 minutes) with a final PCR was ended with one extension cycle (72°C for 10 minutes). The amplified DNA products were resolved by 1% agarose gel electrophoresis and analyzed with an IS-1000 Digital Imaging System (Alpha Innotech Co., San Leandro, CA, USA). The purified PCR products of 002GM1, 002GM2, 002GM3 and 002GM4 were cloned into pEGFP-N1 plasmid (Clontech, Mountain View, CA, USA) and were sequenced to check the integrity of *ORFV002* mutant coding sequences and in-frame cloning with enhanced green fluorescent protein (EGFP).

To construct the flag Flag-tagged MSK1 (pCMVtag2B-MSK1), and mitogen-activated protein kinase kinase 6 (MKK6) (pcDNA3.1-MKK6) and mitogen-activated protein kinase 14 (p38-α) (pcDNA3.1-P38), total RNA was isolated from 293T cells with RNA-Solv Reagent (Omega Bio-tec, Inc., Victoria BC, Canada) in accordance with the manufacturer's instructions. The RNA was reverse transcribed to cDNA using M-MLV Reverse Transcriptase Kit (Promega, Madison, WI, USA) according to the manufacturer's guidelines. Primers were designed for MSK1, MKK6 and p38-α based on the sequences from the database (accession no: NM_004755.2, NM_002758.3 and NM_001315.2):

#### pCMVtag2B-MSK1-Fw

5′-ATATgaattcATGGAGGAGGAGGGTGGCA-3′ (EcoRI).

#### pCMVtag2B-MSK1-Rv

5′-ATATctcgagCTAAGCTACTGAGTCCGAGAAC-3′ (XhoI).

#### pcDNA3.1-MKK6 –Fw

5′-CCCaagcttATGTCTCAGTCGAAAGGCAAG-3′ (Hind III).

#### pcDNA3.1-MKK6-Rv

5′-CCCctcgagTTAGTCTCCAAGAATCAGT-3′ (XhoI).

#### pcDNA3.1-P38-Fw

5′-TATAggatccATGTCTCAGGAGAGGCCCACGT-3′ (BamHI).

#### pcDNA3.1-P38 Rv

5′- ATCActcgagTCAGGACTCCATCTCTTCTTG -3′ (XhoI).

The PCR conditions were used as described above except for the annealing temperature. The purified PCR products were ligated into pCMVtag2B plasmid (Clontech, Mountain View, CA, USA) for Flag-MSK1 and pcDNA3.1 (Life Technologies, Grand Island, NY, USA) for MKK6 and p38-α and sequenced to check the integrity.

### NF-κB Luciferase Reporter Assay

Ovine fetal turbinate (OFTu) cells were cultured in twelve-well plates (1.2×10^5^ cells per well) and co-transfected with the vectors pNF-κB*Luc* (450 ng; Clontech, Mountain View, CA, USA) and pRLTK (50 ng; Promega, Madison, WI, USA) and either pEGFP-N1 (GFP control), or pEGFP-002 (2002GFP, wild type 002-GFP fusion), or one of the ORFV002 deletion mutants fused to EGFP-N1 (pEGFP-002GM1, pEGFP-002GM2, pEGFP-002GM3, or pEGFP-002GM4), using Lipofectamine 2000 (Invitrogen, Carlsbad, CA, USA). The NF-κB luciferase assay was performed as described previously [4]. Statistical analysis of the data was performed by using student's *t* test. Data are expressed as the mean ±SD from three separate experiments.

### Western Blots

OFTu cells were transfected with plasmid pEGFP-N1 (GFP control), pEGFP-002 (wild type ORFV002-GFP fusion) or one of the ORFV002 deletion mutants fused to GFP (pEGFP-002GM1, pEGFP-002GM2, pEGFP-002GM3 or pEGFP-002GM4) as described above, or they were co-transfected with pNF-κB p65 plus pEGFP-N1 (GFP control), pEGFP-002 (wild type ORFV002) or one of the ORFV002 deletion mutants fused to GFP (pEGFP-002GM1, pEGFP-002GM2, pEGFP-002GM3 or pEGFP-002GM4). HEK293T cells were transiently co-transfected with pNF-κB p65, ppCMVtag2B-MSK1, pcDNA3.1-MKK6 and pcDNA3.1-p38-α, plus pEGFP-N1 (GFP control), pEGFP-002 (wild type ORFV002-GFP fusion), or one of the ORFV002 deletion mutants fused to GFP (pEGFP-002GM1, pEGFP-002GM2, pEGFP-002GM3 or pEGFP-002GM4). At 24 hours post-transfection, cells were treated with TNF-α (20 ng/ml) for 0 min, 15 minutes, 30 minutes, 60 minutes or another specified time and were then harvested. A 10% SDS-PAGE gel was prepared and 50 μg of cell lysate was loaded into each well prior to separation. Separated proteins were transferred onto nitrocellulose membranes. Membrane blots were probed with rabbit anti NF-κB-p65 (*p*Ser^276^) (ab30623; abcam, Cambridge, UK), NF-κB-p65 (N-acetyl-Lys^310^) (3045; Cell Signaling Technology, Inc., Danvers, MA, USA), NF-κB-p65 (3034; Cell Signaling Technology, Inc., Danvers, MA, USA), anti-MSK1 (ab63619; abcam, Cambridge, UK), anti-GFP (sc-8334; Santa Cruz Biotechnology, Inc., Santa Cruz, CA, USA) or anti-Flag (F3165; Sigma-ALDRICH, St. Louis, MO, USA) and then followed by incubation with goat-anti-rabbit HRP-conjugated IgG antibody (Santa Cruz Biotechnology, Inc., Santa Cruz, CA, USA) and developed using chemiluminescent substrate ECL (Pierce-Thermo Scientific, Rockford, IL, USA). A densitometric analysis of the blots was performed by using Image J software, version 1.62 (National Institutes of Health, Bethesda, MD, USA). A statistical analysis of the densitometry data was performed by using student's *t* test.

### Confocal Microscopy

OFTu cells were grown on glass cover-slips and co-transfected with pDSRed2-Nuc (Clontech, Mountain View, CA, USA) and either plasmid pEGFP-N1 (GFP control), pEGFP-002 (wild type ORFV002-GFP fusion), or one of the ORFV002 deletion mutants fused to GFP (pEGFP-002GM1, pEGFP-002GM2, pEGFP-002GM3 or pEGFP-002GM4). At 24 hours after transfection, cells were fixed with 4% formaldehyde at room temperature for 1 hour, stained with DAPI (4′, 6-diamidino-2-phenylindole) for 10 minutes and examined by laser scanning confocal microscopy (LSM700; Zeiss, Germany).

## Results

### Comparison of ORFV002 and E1A-12 Amino Acid Sequences and Construction of ORFV002 Mutants

**Figure 1:**
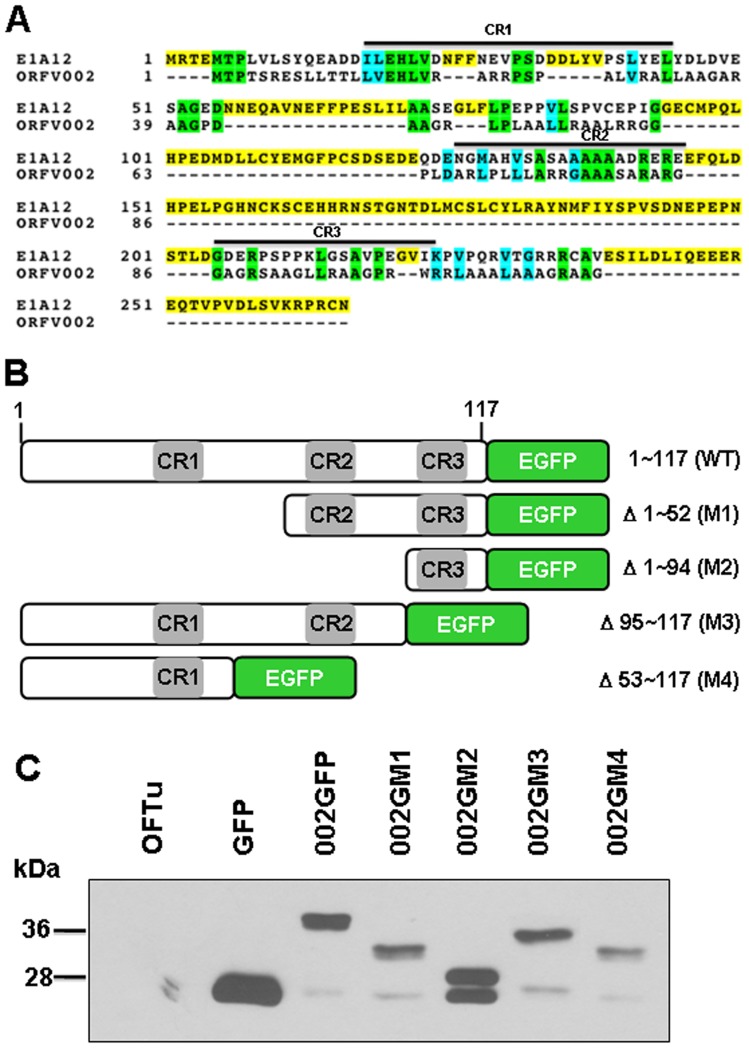
Amino acid sequence comparison between ORFV002 and 12 E1A and ORFV002 mutant construction. A. Comparison of the predicted amino acid sequences of orf virus 002 protein (ORFV002; AY386263.1) and human oncogenic adenovirus type 12 E1A protein (E1A-12; CAA23400.1). These sequences were aligned by using the CLUSTAL W program (San Diego Supercomputer Center Biology Workbench: http://workbench.sdsc.edu/. Solid lines above the alignment indicate three conserved regions (CR1, CR2, and CR3) in E1A-12. B. Construction of ORFV002 wild type and mutants-GFP fusion. C. Western blotting showing the expression of wild type ORFV002GFP and mutants in OFTu cells.

Using the CLUSTAL W program (San Diego Supercomputer Center biology workbench: http://workbench.sdsc.edu/) the predicted amino acid sequences of the orf virus 002 protein (ORFV002; AY386263.1) and human oncogenic adenovirus type 12 E1A protein (E1A-12; CAA23400.1) were compared. We found that the three putative conserved protein domains, CR1, CR2 and CR3 in E1A-12 were also conserved in ORFV002 (Figure 1A). Based on the amino acid sequence alignment between E1A-12 and ORFV002 four plasmids were generated to express deletion mutants of ORFV002 tagged with EGFP designated as: 002GM1 (N-terminal deletion mutant Δ1–52, M1), expressing amino acids 53–117; 002GM2 (N-terminal deletion mutant Δ1–94, M2), expressing amino acids 95–117; 002GM3 (C-terminal deletion mutant Δ95–117, M3), expressing amino acids 1–94; and 002GM4 (C-terminal deletion mutant Δ53–117, M4), expressing amino acids 1–52 (Figure 1B). The nucleotide sequence integrity of each construct was verified by sequencing before use. The total protein levels of wild type and mutants of ORFV002 in OFTu cells were detected by Western blot (Figure 1C).

### Localization of ORFV002 and Its Mutants

**Figure 2:**
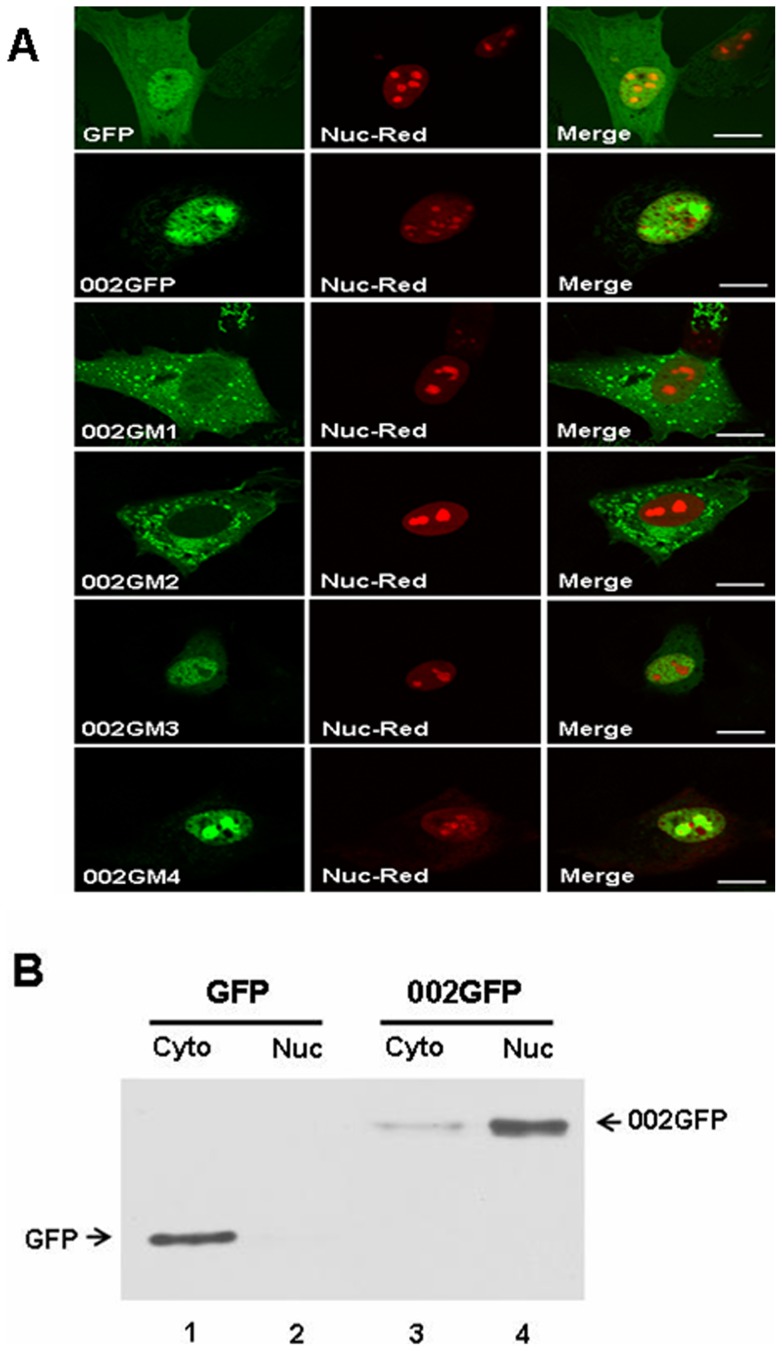
The subcellular localization of ORFV002 and its deletion mutants. A. Confocal microscopy analysis of wild type ORFV002 and its mutants in OFTu cells. Wild type ORFV002, 002GM3 and 002GM4 proteins carried the GFP signal into the nucleus. The GFP signals of 002GM1 and 002GM2 were located in the cytoplasm. Bar: 10 μM. B. Western blot analysis showing ORFV002 mostly located in the nuclear fraction. Cyto: cytoplasmic fraction; Nuc: nuclear fraction

Confocal microscopy showed that wild type ORFV002 expressed in OFTu cells is found mainly in the nucleus, co-localized with Nuc-Red, a nuclear marker (Figure 2A). The signals of the GFP-fusions of ORFV002 C-terminal deletion mutants (002GM3 and 002GM4), were mainly located in the nucleus, while the GFP signals of ORFV002 N-terminal deletion mutants (002GM1 and 002GM2), were mainly located in the cytoplasm (Figure 2A). Western blot analysis confirmed that wild type ORFV002 was mostly located in the nuclear fraction (Figure 2B).

### NF- κB p65 Transcriptional Activity is Inhibited by the N-terminal Region of ORFV002

**Figure 3:**
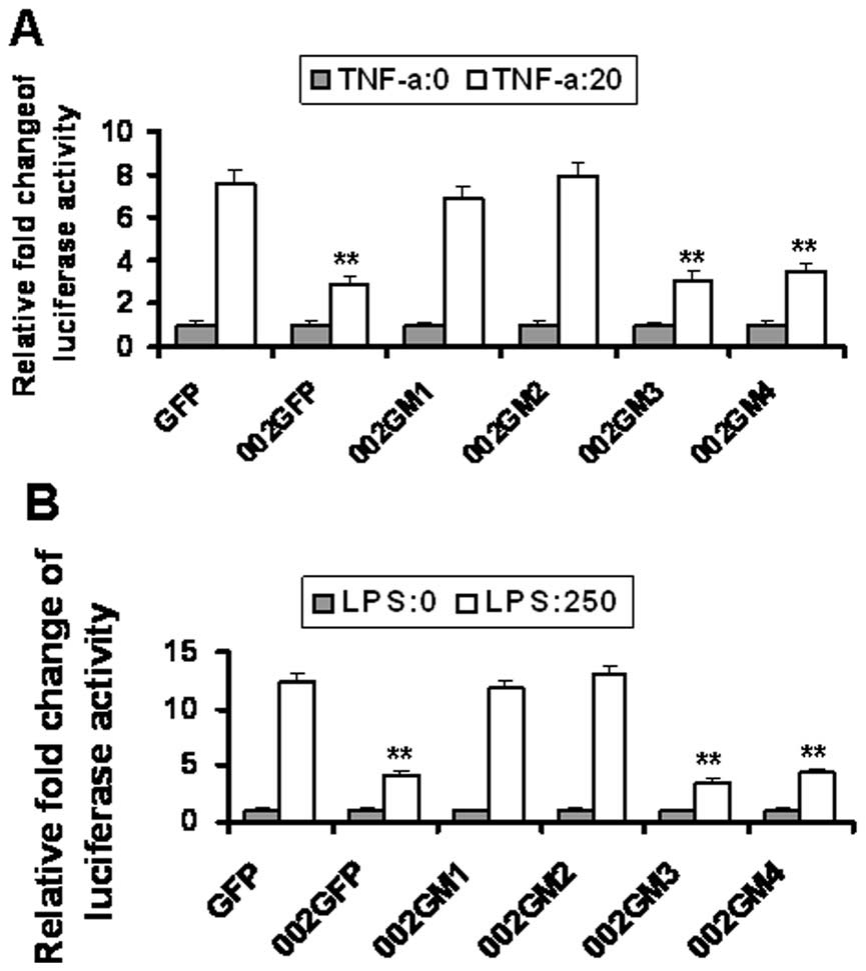
Transcriptional activity by NF-κB p65 is inhibited by the N-terminal region of ORFV002. OFTu cells were co-transfected with a vector encoding a firefly luciferase gene under the control of NF-κB (pNFκB-*Luc*) and with a plasmid encoding sea pansy (*Renilla reniformis*) luciferase under the control of herpesvirus TK promoter (pRL-TK), plus a plasmid expressing GFP (negative control), wild type ORFV002-GFP fusion protein (002GFP), or a ORFV002 deletion mutant fused to GFP (002GM1, 002GM2, pEGFP-002GM3 or 002GM4). At 24 hours after transfection, cells were treated with 20 ng/ml of TNF- α (A) or 250 ng/ml of LPS (B) for 6 hours. Firefly and sea pansy luciferase activities were measured and depicted in the graph as relative fold changes in luciferase activity (**, *P*<0.001). Data are expressed as the mean ± SD from three separate experiments.

OFTu cells were co-transfected with a vector encoding a firefly luciferase gene under the control of a NF-κB-inducible promoter (pNFκBB-*Luc*) and with a plasmid encoding sea pansy (*Renilla reniformis*) luciferase under the control of herpesvirus TK promoter (pRL-TK), and additionally co-transfected with pEGFP002 or one of the plasmids expressing an ORFV002 deletion mutant (002GM1, 002GM2, 002GM3, or 002GM4). After transfection for 24 hours cells were treated with 20 ng/mL of TNF- α or 250 ng/mL of lipopolysaccharide (LPS) for 6 hours. Firefly and sea pansy luciferase activities were measured and expressed as relative fold changes in luciferase activity. The fold-changes increases in luciferase activity indicate that ORFV002, and the intact N-terminus of ORFV002 (002GM3 and 002GM4) significantly inhibited transcriptional activity by NF-κB p65 as compared with GFP control or the intact C-Terminus of ORFV002, as demonstrated by 002GM1 and 002GM2 (Figure 3).

### ORFV002 and Its N-terminus Prevent NF-κB p65 Lys^310^ Acetylation

**Figure 4:**
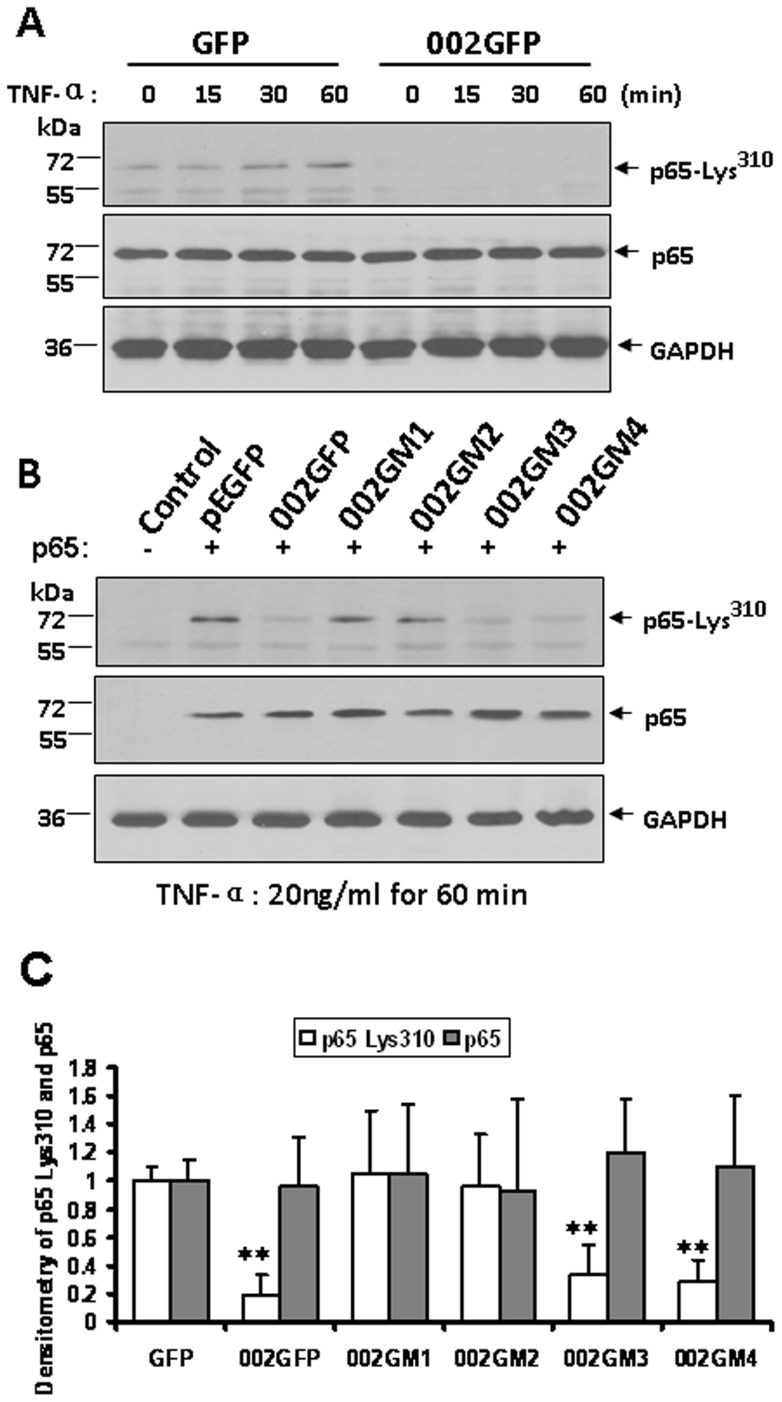
Wild type ORFV002 and its mutants in OFTu cells prevent NF-κB p65 (Lys^310^) acetylation. A. OFTu cells were co-transfected with plasmid pNF-κB p65 and either pEGFP-N1 or pORFV002-EGFP. At 24 hours post transfection, cells were treated with 20 ng/ml of TNF-α for 0, 15, 30 and 60 minutes and harvested at the indicated times. Total cell lysis protein extracts (50 μg) were loaded in 10 percent SDS-PAGE gel, blotted, and probed with antibodies against NF-κB p65 (Lys^310^) (top), NF-κB p65 (middle), or GAPDH (bottom). B. OFTu cells were co-transfected with plasmid pNF-κB p65 and ORFV002 mutants. At 24 hours post transfection, cells were treated with 20 ng/ml of TNF-α for 60 minutes. Total cell lysates were detected by WB similar to A. C. Densitometry of NF-κB-p65 (acety-Lys^310^) bands normalized to loading control GAPDH (**, *P<*0.005). The results are representative of three independent experiments.

We showed previously that ORFV002 inhibits the acetylation of NF-κB p65. In order to define the region of ORFV002 responsible for inhibiting the acetylation of NF-κB p65, different N- and C-terminal truncation mutants of ORFV002 were generated (Figure 1B). OFTu cells were co-transfected with a pNFκB p65 plasmid and a plasmid expressing GFP alone (negative control), wild type ORFV002-GFP fusion protein or one of the ORFV002 deletion mutants fused to GFP. The acetylation Lys^310^ was examined by Western blotting using an anti-NF-κB p65 (N-acetyl-Lys^310^) antibody (Figure 4). As expected from our previous studies [4] the acetylation of NF-κB p65 (Lys^310^) was dramatically reduced in the presence of wild type 002GFP (Figure 4A and 4B, lane 3). As with wild type ORFV002, the intact N-terminus of ORFV002 (002GM3 and 002GM4) also induced a significant reduction of p65 Lys^310^ acetylation (Figure 4B, lanes 6 and 7). In contrast, deletion of the first 52 residues of ORFV002 (Δ1–52), which contains CR1, or deletion of the first 94 residues of ORFV002 (Δ1–94), which contains CR1 and CR2 (see Figure 1A and 1B), abolished the ability of ORFV002 to inhibit NF-κB p65 (Lys^310^) acetylation (Fig. 4B, lanes 4 and 5). The total protein levels of p65 and GAPDH expressed in OFTu cells were comparable (Figure 4). These results indicate that the ORFV002 N-terminus (residues 1–52) mediates NF-κB p65 acetylation at Lys^310^ as effectively as the whole ORFV002 protein.

### ORFV002 and its N-terminus Prevent NF-κB p65 (Ser^276^) Phosphorylation

**Figure 5:**
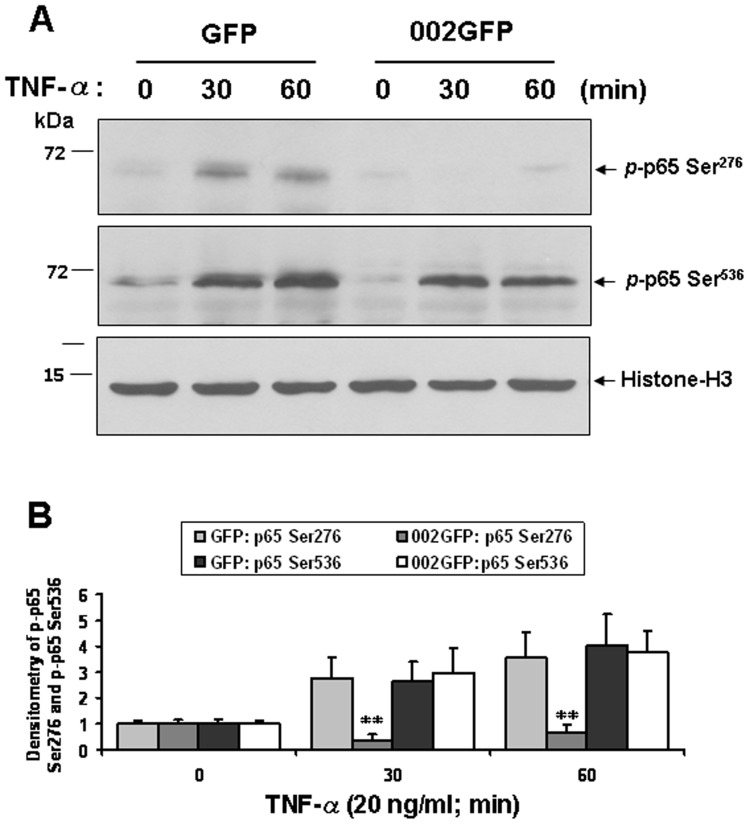
ORFV002 prevents NF-κB-p65 Ser276 phosphorylation. A. OFTu cells were co-transfected with plasmid pNF-κB-p65 and either pEGFP-N1 or pORFV002-EGFP. At 24 hours post transfection, cells were treated with 20 ng/ml of TNF-α for 0, 30 and 60 minutes and harvested at the indicated times. Nuclear protein fractions were extracted. Protein extracts (50 μg) were resolved by SDS-PAGE, blotted, and probed with antibodies against p-NF-κB-p65-Ser^276^ (top), p-NF-κB-p65-Ser^536^ (middle), or histone H3 (bottom). B. Densitometry of p-NF-κB-p65 Ser^276^ and Ser^536^ bands normalized to loading control histone H3 (**, P<0.005). The results are representative of three independent experiments.

**Figure 6:**
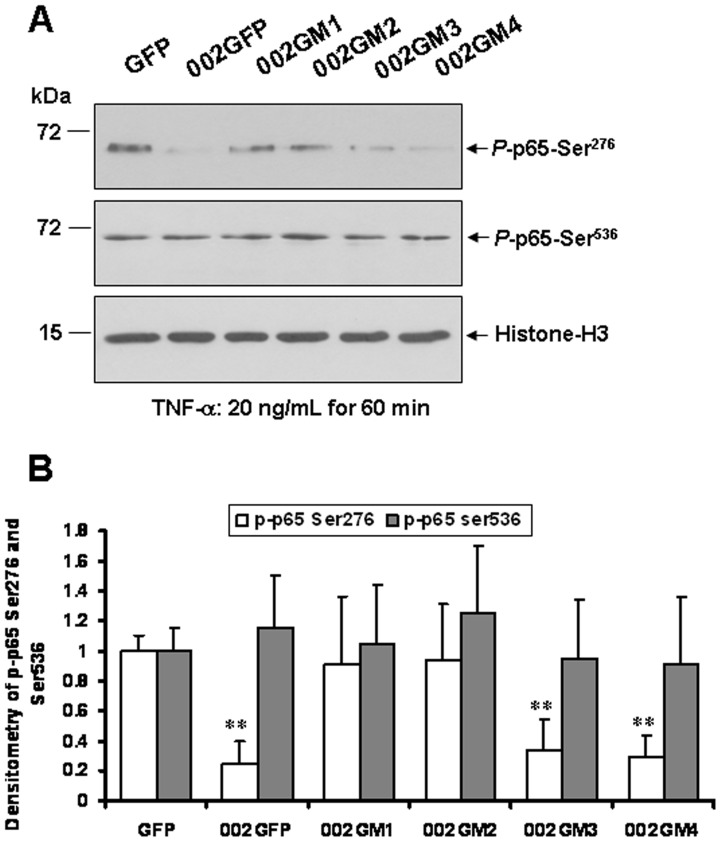
The N-terminus of ORFV002 blocks NF-κB p65 (Ser^276^) phosphorylation. A. OFTu cells were co-transfected with plasmid pT7-NF-κB-p65 and pEGFP-N1, wild type ORFV002GFP, or mutant ORFV002GFP plasmids, treated with TNF-α (20 ng/ml), and harvested at 60 minutes after TNF-α treatment. Nuclear protein fractions were extracted and western blot analysis was performed as described above. B. Densitometry of *p*-p65-Ser^276^ bands normalized to loading control histone H3 (**, *P<*0.005). The results are representative of three independent experiments.

To investigate further whether ORFV002 affects NF-κB p65 (Ser^276^) phosphorylation, OFTu cells were co-transfected with plasmid pNF-κB p65 and either pEGFP-N1 or p002-EGFP or deletion of mutant constructs. At 24 hours after transfection cells were treated with 20 ng/ml of TNF-α for 0, 30 and 60 minutes. After the cells were harvested, the nuclei protein was extracted. The phosphorylation of NF-κB p65 (Ser^276^) of the nuclei protein was examined by Western blotting using an anti-NF-κB p65 (pSer^276^) antibody, while phosphorylation of NF-κB p65 at Ser^536^ was detected by immunoblotting using an anti-NF-κB p65 (pSer^536^) antibody (Figure 5). The Western blotting results showed that ORFV002 significantly inhibits phosphorylation of NF-κB p65 Ser^276^ but not NF-κB p65 Ser^536^ (Figure 5A and 5B). As with wild type ORFV002, mutants of 002GM3 and M4 also induced significant reduction of NF-κB p65 Ser^276^ phosphorylation (Figure 6, lanes 6 and 7). In contrast, deletion of the N-terminal 52 residues (CR1) or 94 residues (CR1+CR2) abolished the ability of ORFV002 to inhibit p65 Ser^276^ phosphorylation (Figure 6B, lanes 4 and 5). The total protein levels of phospho-p65 and histone-H3 expressed in OFTu cells were comparable (Figure 6). These results indicate that the N-terminus of ORFV002 (residues 1 to 52) mediates p65 phosphorylation at Ser^276^.

### ORFV002 Interferes With NF-κB-p65 Ser^276^ Phosphorylation Induced by MSK1

**Figure 7:**
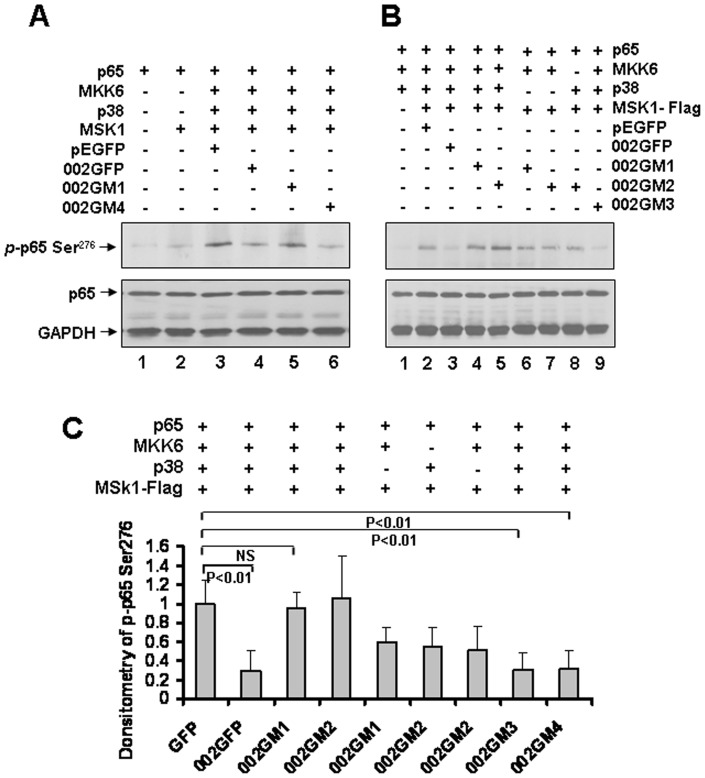
ORFV002 interferes with NF-κB p65 (Ser^276^) phosphorylation induced by MSK1. A. HEK293T cells were transfected using expression vectors for p65, Flag-MSK1 plus GFP, 002GFP, 002GM1 or 002GM3. Activation of MSK1 was achieved by co-transfecting MKK6 and p38 kinase. At 24 hours post-transfection, cells were treated with 20 ng/ml TNF-α for 30 minutes, and cell lysates were prepared and detected by immunoblotting with an anti-NF-κB-p65 (*p*Ser^276^) antibody. B. HEK293T cells were co-transfected with plasmid p65 plus: lane 1: MKK6 + p38; lane 2: MKK6 + p38 + MSK1 + pEGFP; lane 3: MKK6 + p38 + MSK1 + 002GFP; lane 4: MKK6 + p38 + MSK1 + 002GM1; lane 5: MKK6 + p38 + MSK1 + 002GM2; lane 6: MKK6 + MSK1 + 002GM1; lane 7: p38 + MSK1 + 002GM2; lane 8: MKK6 + MSk1 + 002GM2; lane 9: MKK6 + p38 + MSK1 + 002GM3. At 24 hours post-transfection, cells were treated with 20 ng/ml TNF-α for 30 minutes, and cell lysates were prepared and detected by immunoblotting with an anti-NF-κB-p65 (*p*Ser^276^) phosphorylation antibody antibody. C. The relative densitometry of NF-κB p65 (*p*Ser^276^) phosphorylation bands normalized to the level for loading control (GAPDH). The results are representative of three independent experiments. NS: No significant difference.

To investigate further whether ORFV002 affects production of NF-κB p65 (pSer^276^) by MSK1, HEK293T cells were transiently transfected using expression vectors for p65, Flag-MSK1 plus GFP, 002GFP, 002GM1, or 002GM3 with pcDND3.1-MKK6 and pcDNA3.1-P38. At 24 hours post-transfection, cells were treated with 20 ng/ml TNF-α for 30 minutes, and cell lysates were prepared and phosphorylation of NF-κB p65 at Ser^276^ was detected by immunoblotting using an anti-NF-κB p65 (*p*Ser^276^) antibody (Figure 7). The western blotting results showed that nuclear kinase MSK1, when activated by TNF-α, specifically phosphorylated NF-κB p65 at the serine residue in position 276 (*p*Ser^276^) and this phosphorylation was inhibited by ORFV002, or by the intact N-terminus of ORFV002 (002GM3 and 002GM4). Expression of NF-κB p65 (*p*Ser^276^) was further enhanced by TNF-α treatment when cells were co-transfected with MKK6 and p38 in addition to NF-κB p65 and MSK1 (Figure 7A, line 3 and B, lane 2), whereas co-transfection of NF-κB p65 and MSK1 with MKK6 or p38 alone resulted in relatively low levels of NF-κB p65 Ser^276^ phosphorylation (Figure 7B, lane 6, 7 and 8). Very low level of NF-κB p65 Ser^276^ phosphorylation was detected when cells were transfected with NF-κB p65 alone (Figure 7A, lane 1), NF-κB p65 plus MSK1 (Figure 7A, lane 2), or NF-κB p65 plus MKK6 and p38 (Figure 7B, lane 1 and C) suggesting that MKK6 and p38 were absolutely required for MSK1 activation and NF-κB-driven gene transcription in response to TNF-α. GFP-fusions of wild type ORFV002 (002GFP) and the intact N-terminus of ORFV002 (002GM3 and 002GM4) significantly inhibited NF-κB p65 Ser^276^ phosphorylation (Figure 7A, lane 4 and 6; B, lane 3 and 9), which is consistent with previous observations [20], [21]. The results indicate that p38 and ERK mitogen-activated protein kinases (MAPKs) are absolutely required for full activation of MSK1 activity and NF-κB-driven gene transcription in response to TNF- α.

### ORFV002 Interferes With the Interaction Between NF-κB p65 and MSK1 *in vivo*

**Figure 8:**
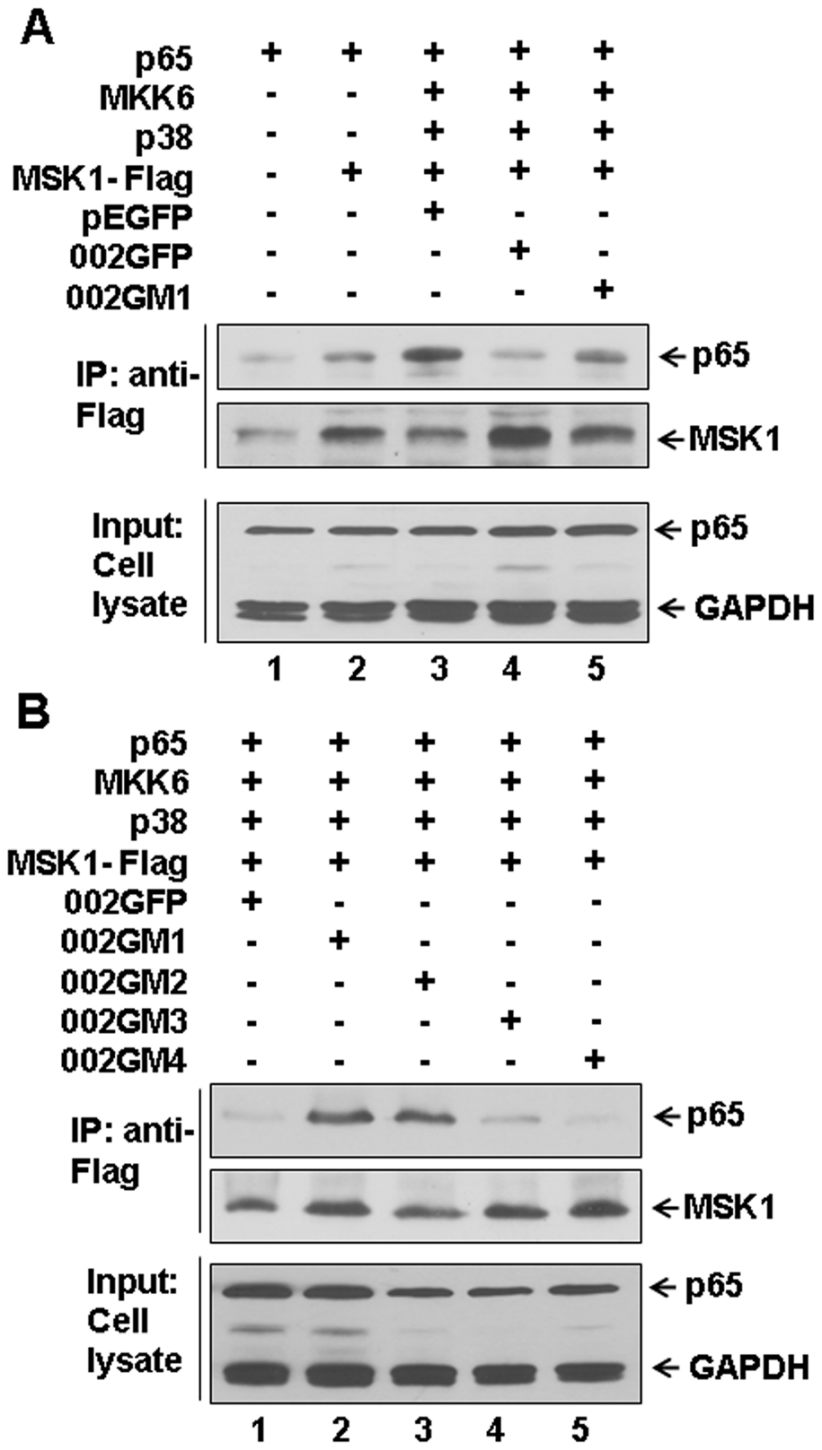
ORFV002 inhibits the interaction between NF-κB p65 and MSK1 *in vivo*. HEK293T cells were transiently transfected using expression vectors for NF-κB p65, Flag-MSK1 plus GFP, 002GFP or 002GM1-4 (A and B). Activation of MSK1 was achieved by co-transfecting MKK6 and p38 kinase. At 24 hours post-transfection, cells were treated with 20 ng/ml TNF-α for 30 minutes, and cell lysates were prepared and subject to immunoprecipitation with anti-Flag-MSK1 antibody. Co-precipitating p65 was detected by immunoblotting with an anti-p65 antibody. To monitor the activation status of MSK1, blots were stripped and reprobed using an anti- MSK1 antibody.

To investigate the role of ORFV002 in the interaction of NF-κB p65 and MSK1, HEK293T cells were transiently transfected using expression vectors for NF-κB p65, Flag-MSK1 plus GFP, or 002GFP or 002GM1 with expression vectors for MKK6 and p38. At 24 hours post-transfection cells were treated with 20 ng/ml TNF-α for 30 minutes and cell lysates were prepared for immunoprecipitation with an anti-Flag-MSK1 antibody. Co-precipitating NF-κB p65 was detected by immunoblotting using an anti- NF-κB p65 antibody. To monitor the activation status of MSK1 blots were stripped and re-probed using an anti-MSK1 antibody. The results showed that the MSK1 and NF-κB p65 indeed interact and that ORFV002 and the intact N-terminus of ORFV002 (002GM3 and 002GM4) interfere with the interaction between NF-κB p65 and MSK1 (Figure 8A, lane 4 and B lane 1, 4 and 5). There was no significant effect of 002GM1 and 002GM2 on the interaction between NF-κB p65 and MSK1 as compared to GFP alone, a negative control (Figure 8A, lanes 3 and 5; or 8B, lanes 2 and 3).

## Discussion

Previous studies showed that ORFV002 inhibits the activation of NF-κB, the master transcriptional regulator of immune response during ORFV infection [4]. In this study the luciferase assay showed that ORFV002 mutants lacking the N-terminus did not inhibit activation of NF-κB, while wild type ORFV002 and deletion mutants of ORFV002 retaining an intact N-terminus did inhibit activation of NF-κB. This result confirmed that the N-terminus of ORFV002 is important for inhibiting NF-κB activation.

The post-translational modifications of NF-κB, including site-specific phosphorylation and acetylation, are important for the optimal transactivation activity of NF-κB [13], [14]. In this study we revealed that the N-terminus of ORFV002 inhibits NF-κB from stimulating transcription by blocking phosphorylation of NF-κB p65 Ser^276^ but not Ser^536^. Usually NF-κB p65 is phosphorylated by the protein kinase A (PKA) upon lipopolysaccharide (LPS) triggering [19] or mitogen- and stress-activated kinase-1 (MSK1) after TNF-stimulation [20]. In our research, we transfected MKK6 and p38 kinases and used TNF-α to activate MSK1 in HEK293T cells. The HEK293T cells were co-transfected with NF-κB p65, and GFP, 002GFP, 002GM1 or 002GM4, plus plasmids expressing MKK6 and p38 to investigate the role of ORFV002 on MSK1 and status of p65 Ser^276^ phosphorylation. The results showed that NF-κB p65 is phosphorylated at Ser^276^ by MSK1 after TNF-α stimulation, which is consistent with previous studies [20], [22]–[24] and ORFV002, and its C-terminal deletion mutants 002GM3 and 002GM4, significantly inhibited the activation of NF-κB p65 through MSK1. Our results also showed that ORFV002 interfered with the interaction between NF-κB p65 and MSK1. Based on these results, ORFV002 inhibited NF-κB p65 phosphorylation by a competitive inhibition of interactions between MSK1 and NF-κB p65 by ORFV002, which further inhibited the acetylation of NF-κB p65 at Lys^310^.

It has been confirmed that phosphorylated NF-κB p65 could recruit the transcriptional co-activators such as p300 and CBP to the NF-κB-bound promoter and facilitate DNA binding ability of NF-κB p65 [15]–[18]. Also, through its N-terminal regions, ORFV002 inhibits phosphorylation of NF-κB p65 at the Ser^276^ site, interfering with interactions with other transcriptional co-activators, such as p300 and CBP. As shown in the previous report, ORFV002 can physically interact with NF-κB p65 [4]. It is possible that the N-terminus of ORFV002 affects NF-κB p65 (Lys^310^) acetylation through blocking phosphorylation of NF-κB p65 (Ser^276^) and preventing NF-κB-dependent transcriptional activation. Furthermore, confocal microscopy analysis of ORFV002 mutants and the detection of low levels of NF-κB p65 (pSer^276^) in nuclear extracts of cells expressing ORFV002 deletion mutants retaining an intact N-terminus confirm that the N-terminus of ORFV002 is sufficient to inhibit phosphorylation of NF-κB p65 at Ser^276^ in the nucleus.

Many studies have shown that nuclear kinase MSK1 is a candidate for phosphorylation of NF-κB p65 at Ser^276^, which acts downstream of p38 and ERK MAPKs in response to TNF-α, associates with NF-κB p65 in a stimulus-dependent manner and specifically phosphorylates the serine residue at position 276, thus leading to its positioning at NF-κB-containing promoter sections and selective stimulation of particular NF-κB-driven genes [20], [21], [22]. In this study we present the first data to show that ORFV002 and its N-terminus inhibit NF-κB p65 Ser^276^ phosphorylation by MSK1 activation pathway. Levels of NF-κB p65 phosphorylated at Ser^276^ was increased by TNF-α treatment when NF-κB p65 and MSK1 were co-transfected with MKK6 and p38 (Figure 7A, line 3 and B, lane 2), whereas phosphorylation of NF-κB p65 at Ser^276^ was only partially increased when p65 and MSK1 were co-transfected with MKK6 or p38 alone (Figure 7B, lane 6, 7 and 8), suggesting that MKK6 and p38 were required for full activation of MSK1 and NF-κB-driven gene transcription in response to TNF-α. These results are consistent with previous observations [20], [23], [24].

**Figure 9:**
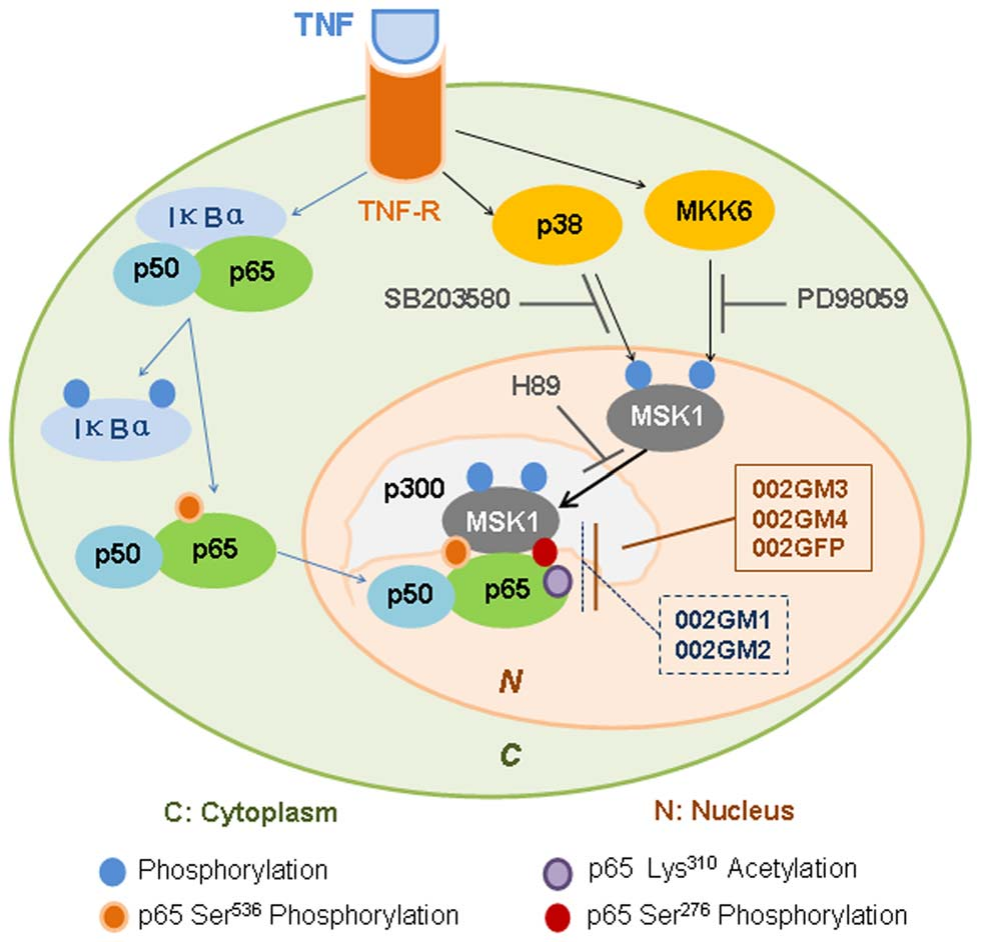
Overview of the phosphorylation pathways inhibits by ORFV002. Binding of TNF to its receptor (TNF-R) causes TNF-R to phosphorylate IκBα, and NF-κB p65 at Ser^536^, freeing NF-κB p50/p65 (pSer^536^) from IκBα, and allowing NF-κB p50/p65 (pSer^536^) to translocate to the nucleus. After stimulation by TNF p38 and ERK MAPK kinases were also activated, both of which can activate MSK1 independently, with maximal activation requiring phosphorylation of MSK1 by both kinases. In the nucleus, activated MSK1 phosphorylates NF-κB p65 at Ser^276^, resulting in fully functional NF-κB that can interact with other transcriptional activators, such as p300, to drive transcription of NF-κB-regulated genes. Wild type ORFV002 (002GFP) and C-terminal deletion mutants of ORFV002 (002GM3 and 002GM4) can block MSK1 phosphorylation of NF-κB p65 at Ser^276^, while N-terminal deletion mutants of ORFV002 (002GM1 and 002GM2) cannot. SB203580, PD98059, and H89 are inhibitors of p38 activation of MSK1, MAPK activation of MSK1 and the ability of activate MSK1 to phosphorylate NF-κB p65, respectively (See Ref [20]).

Taken together, a model of the phosphorylation pathways inhibited by ORFV002 is presented in Figure 9, in which cytoplasmic NF- κB activation is followed by nuclear phosphorylation of the p65 subunit at Ser^276^. Binding of TNF to its receptor (TNF-R) causes TNF-R to phosphorylate IκBα, and NF-κB p65 at Ser^536^, freeing NF-κB p50/p65 (pSer^536^) from IκBα, and allowing NF-κB p50/p65 (pSer^536^) to translocate to the nucleus. Stimulation by TNF also activates p38 kinase and ERK MAPK kinases, both of which can activate MSK1 independently, with maximal activation requiring phosphorylation of MSK1 by both kinases. In the nucleus, activated MSK1 phosphorylates NF-κB p65 at Ser^276^, resulting in fully functional NF-κB that can interact with other transcriptional activators, such as p300, to drive transcription of NF–κB-regulated genes. Wild type ORFV002 (002GFP) and C-terminal deletion mutants of ORFV002 (002GM3 and 002GM4) can block MSK1 phosphorylation of NF-κB p65 at Ser^276^, while N-terminal deletion mutants of ORFV002 (002GM1 and 002GM2) cannot. SB203580, PD98059, and H89 are inhibitors of p38 activation of MSK1, MAPK activation of MSK1 and the ability of activate MSK1 to phosphorylate NF-κB p65, respectively (See Ref [20]).

In conclusion the N-terminus of ORFV002 can inhibit phosphorylation of NF- κB p65 Ser^276^ by weakening the interaction of MSK1 and NF-κB p65 and by blocking the acetylation of NF-κB p65. This is the first identification of a novel function of ORFV002 protein, in which it can inhibit the NF-κB signaling pathway through its N-terminus (residues 1 to 52).

## References

[1] Vikøren T, Lillehaug A, Akerstedt J, Bretten T, Haugum M, et al (2008) A severe outbreak of contagious ecthyma (orf) in a free-ranging musk ox (*Ovibos moschatus*) population in Norway. Vet Microbiol 127: 10–20. 17768017 10.1016/j.vetmic.2007.07.029

[2] Al-Salam S, Nowotny N, Sohail MR, Kolodziejek J, Berger TG (2008) Ecthyma contagiosum (orf)-report of a human case from the United Arab Emirates and review of the literature. J Cutan Pathol 35: 603–607. 18201239 10.1111/j.1600-0560.2007.00857.x

[3] McElroy MC, Bassett HF (2007) The development of oral lesions in lambs naturally infected with orf virus. Vet J 174: 663–664. 17185014 10.1016/j.tvjl.2006.10.024

[4] Diel DG, Luo S, Delhon G, Peng Y, Flores EF, et al (2011) A nuclear inhibitor of NF-kappaB encoded by a poxvirus. J Virol 85: 264–275. 20980501 10.1128/JVI.01149-10 PMC3014193

[5] Mercer AA, Ueda N, Friederichs SM, Hofmann K, Fraser KM, et al (2006) Comparative analysis of genome sequences of three isolates of Orf virus reveals unexpected sequence variation. Virus Res 116: 146–158. 16274827 10.1016/j.virusres.2005.09.011

[6] Delhon G, Tulman ER, Afonso CL, Lu Z, de la Concha-Bermejillo A, et al (2004) Genomes of the parapoxviruses ORF virus and bovine papular stomatitis virus. J Virol 78: 168–177. 14671098 10.1128/JVI.78.1.168-177.2004 PMC303426

[7] Diel DG, Luo S, Delhon G, Peng Y, Flores EF, et al (2011) Orf virus ORFV121 encodes a novel inhibitor of NF-kappa B that contributes to virus virulence. J Virol 85: 2037–2049. 21177808 10.1128/JVI.02236-10 PMC3067802

[8] Diel DG, Delhon G, Luo S, Flores EF, Rock DL (2010) A novel inhibitor of the NF-{kappa}B signaling pathway encoded by the parapoxvirus orf virus. J Virol 84: 3962–3973. 20147406 10.1128/JVI.02291-09 PMC2849485

[9] Perkins ND (2006) Post-translational modifications regulating the activity and function of the nuclear factor kappa B pathway. Oncogene 25: 6717–6730. 17072324 10.1038/sj.onc.1209937

[10] Guan H, Jiao J, Ricciardi RP (2008) Tumorigenic adenovirus type 12 E1A inhibits phosphorylation of NF-kappaB by PKAc, causing loss of DNA binding and transactivation. J Virol 82: 40–48. 17959673 10.1128/JVI.01579-07 PMC2224381

[11] Jiao J, Guan H, Lippa AM, Ricciardi RP (2010) The N terminus of adenovirus type 12 E1A inhibits major histocompatibility complex class I expression by preventing phosphorylation of NF-kappaB p65 Ser276 through direct binding. J Virol 84: 7668–7674. 20504937 10.1128/JVI.02317-09 PMC2897633

[12] McClurkin AW, Pirtle EC, Coria MF, Smith RL (1974) Comparison of low- and high-passage bovine turbinate cells for assay of bovine viral diarrhea virus. Arch Gesamte Virusforsch 45: 285–289. 4472511 10.1007/BF01249692

[13] Joo JH, Jetten AM (2008) NF-kappaB-dependent transcriptional activation in lung carcinoma cells by farnesol involves p65/RelA (Ser276) phosphorylation via the MEK-MSK1 signaling pathway. J Biol Chem 283: 16391–16399. 18424438 10.1074/jbc.M800945200 PMC2423266

[14] Huang B, Yang XD, Lamb A, Chen LF (2010) Posttranslational modifications of NF-kappaB: another layer of regulation for NF-kappaB signaling pathway. Cell Signal 22: 1282–1290. 20363318 10.1016/j.cellsig.2010.03.017 PMC2893268

[15] Zhong H, Voll RE, Ghosh S (1998) Phosphorylation of NF-kappa B p65 by PKA stimulates transcriptional activity by promoting a novel bivalent interaction with the coactivator CBP/p300. Mol Cell 1: 661–671. 9660950 10.1016/s1097-2765(00)80066-0

[16] Chen LF, Fischle W, Verdin E, Greene WC (2001) Duration of nuclear NF-kappaB action regulated by reversible acetylation. Science 293: 1653–1657. 11533489 10.1126/science.1062374

[17] Gao N, Asamitsu K, Hibi Y, Ueno T, Okamoto T (2008) AKIP1 enhances NF-kappaB-dependent gene expression by promoting the nuclear retention and phosphorylation of p65. J Biol Chem 283: 7834–7843. 18178962 10.1074/jbc.M710285200

[18] Dong J, Jimi E, Zhong H, Hayden MS, Ghosh S (2008) Repression of gene expression by unphosphorylated NF-?B p65 through epigenetic mechanisms. Genes Dev 22: 1159–1173. 18408078 10.1101/gad.1657408 PMC2335313

[19] Zhong H, SuYang H, Erdjument-Bromage H, Tempst P, Ghosh S (1997) The transcriptional activity of NF-?B is regulated by the I? B-associated PKAc subunit through a cyclic AMP-independent mechanism, Cell 89: 413–424. 10.1016/s0092-8674(00)80222-6 9150141

[20] Vermeulen L, De Wilde G, Van Damme P, Vanden Berghe W, Haegeman G (2003) Transcriptional activation of the NF-kappaB p65 subunit by mitogen- and stress-activated protein kinase-1 (MSK1). EMBO J 22: 1313–1324. 12628924 10.1093/emboj/cdg139 PMC151081

[21] Deak M, Clifton AD, Lucocq LM, Alessi DR (1998) Mitogen- and stress-activated protein kinase-1 (MSK1) is directly activated by MAPK and SAPK2/p38, and may mediate activation of CREB. EMBO J 17(15): 4426–4441. 9687510 10.1093/emboj/17.15.4426 PMC1170775

[22] Reber L, Vermeulen L, Haegeman G, Frossard N (2009) Ser276 phosphorylation of NF-kB p65 by MSK1 controls SCF expression in inflammation. PLoS One 4: e4393. 19197368 10.1371/journal.pone.0004393 PMC2632887

[23] Vanden Berghe W, Plaisance S, Boone E, De Bosscher K, Schmitz ML, et al (1998) p38 and extracellular signal-regulated kinase mitogen-activated protein kinase pathways are required for nuclear factor-kappaB p65 transactivation mediated by tumor necrosis factor. J Biol Chem 273(6): 3285–3290. 9452444 10.1074/jbc.273.6.3285

[24] Thomson S, Clayton AL, Hazzalin CA, Rose S, Barratt MJ, et al (1999) The nucleosomal response associated with immediate-early gene induction is mediated via alternative MAP kinase cascades: MSK1 as a potential histone H3/HMG-14 kinase. EMBO J 18(17): 4779–4793. 10469656 10.1093/emboj/18.17.4779 PMC1171550

